# Bis[μ-*N*-(2-oxidobenzyl­idene)pyridine-2-carbohydrazidato]bis­[chlorido(methanol-κ*O*)erbium(III)]

**DOI:** 10.1107/S1600536812013979

**Published:** 2012-04-13

**Authors:** Hua Yang

**Affiliations:** a College of Chemistry and Chemical Engineering, Yan’an University, Yan’an, Shaanxi 716000, People’s Republic of China

## Abstract

In the binuclear title complex, [Er_2_(C_13_H_9_N_3_O_2_)_2_Cl_2_(CH_3_OH)_2_], the entire mol­ecule is generated by the application of inversion symmetry. Each Er^III^ ion is seven-coordinated by two O atoms and one N atom from one *N*-(2-oxidobenzyl­idene)pyridine-2-carbohydrazidate (*L* ^2−^) ligand, one O atom and one N atom from the symmetry-related *L* ^2−^ ligand, one O atom of a methanol mol­ecule and one chloride anion. The coordination geometry is based on a pseudo-penta­gonal bipyramid. Linear supra­molecular chains along [010] are formed in the crystal packing through O—H⋯Cl hydrogen bonds.

## Related literature

For complexes containing salicyl­aldehyde-2-pyridine­carboxyl-hydrazone and related ligands, see: Guo *et al.* (2011*a* ▶,*b* ▶); Bai *et al.* (2005 ▶, 2006 ▶); Wu *et al.* (2004 ▶); Milway *et al.* (2003 ▶). For the mechanism of the hydrolysis of salicyl­aldehyde thio­semicarbazone, see: Narang & Aggarwal (1974 ▶).
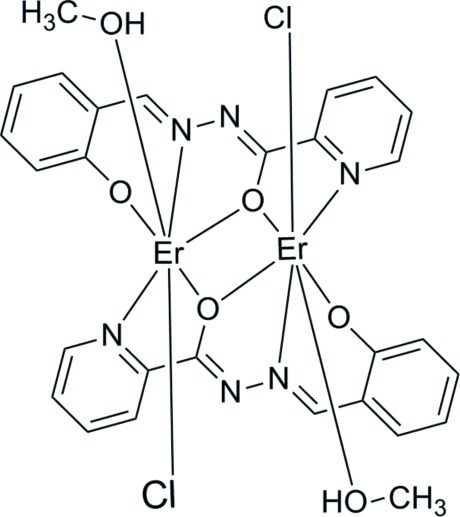


## Experimental

### 

#### Crystal data

[Er_2_(C_13_H_9_N_3_O_2_)_2_Cl_2_(CH_4_O)_2_]
*M* *_r_* = 947.97
Monoclinic, 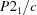

*a* = 9.5810 (4) Å
*b* = 7.0906 (3) Å
*c* = 22.3504 (8) Å
β = 96.920 (3)°
*V* = 1507.31 (10) Å^3^
*Z* = 2
Mo *K*α radiation
μ = 5.76 mm^−1^
*T* = 128 K
0.15 × 0.13 × 0.12 mm

#### Data collection

Bruker SMART CCD diffractometer
Absorption correction: multi-scan (*SADABS*; Bruker 2000 ▶) *T*_min_ = 0.479, *T*_max_ = 0.545
14182 measured reflections
3732 independent reflections
2931 reflections with *I* > 2σ(*I*)
*R*_int_ = 0.040

#### Refinement

*R*[*F* ^2^ > 2σ(*F* ^2^)] = 0.027
*wR*(*F* ^2^) = 0.059
*S* = 0.99
3732 reflections
200 parameters
H-atom parameters constrained
Δρ_max_ = 1.25 e Å^−3^
Δρ_min_ = −0.71 e Å^−3^

### 

Data collection: *SMART* (Bruker, 2000 ▶); cell refinement: *SAINT* (Bruker, 2000 ▶); data reduction: *SAINT*; program(s) used to solve structure: *SHELXS97* (Sheldrick, 2008 ▶); program(s) used to refine structure: *SHELXL97* (Sheldrick, 2008 ▶); molecular graphics: *SHELXTL* (Sheldrick, 2008 ▶); software used to prepare material for publication: *SHELXTL*.

## Supplementary Material

Crystal structure: contains datablock(s) I, global. DOI: 10.1107/S1600536812013979/tk5069sup1.cif

Structure factors: contains datablock(s) I. DOI: 10.1107/S1600536812013979/tk5069Isup2.hkl

Additional supplementary materials:  crystallographic information; 3D view; checkCIF report

## Figures and Tables

**Table 1:** Selected bond lengths (Å)

Er1—O1	2.157 (3)
Er1—O2^i^	2.284 (3)
Er1—O2	2.316 (3)
Er1—O3	2.327 (3)
Er1—N3	2.433 (3)
Er1—N1	2.488 (3)
Er1—Cl1	2.5901 (12)

Symmetry code: (i) 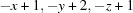.

**Table 2:** Hydrogen-bond geometry (Å, °)

*D*—H⋯*A*	*D*—H	H⋯*A*	*D*⋯*A*	*D*—H⋯*A*
O3—H7⋯Cl1^ii^	0.95	2.42	3.128 (4)	131

Symmetry code: (ii) 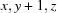.

**Fig. 1.**
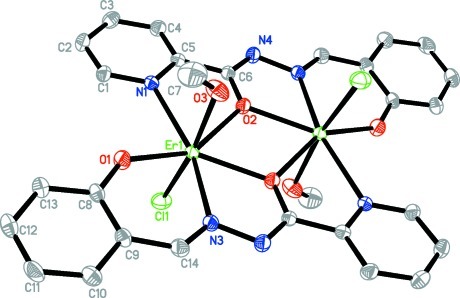
The molecular structure of the title compound, with atom labels and 30% probability displacement ellipsoids. The H atoms have been omitted for clarity.

**Fig. 2.**
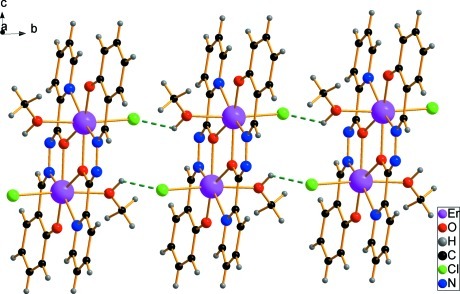
View of the linear supramolecular chain along [010] with the O—H···Cl hydrogen bonds shown as dashed lines.

## supplementary crystallographic information

### Comment

The chemistry of coordination complexes supported by salicylaldehyde-2-pyridinecarboxyl-hydrazone (H_2_L) and its derivatives has received intensive attention as these form coordination complexes with aesthetically pleasing structures and intriguing magnetic behaviour (Guo *et al.*, 2011*a*,*b*). A handful of transition metal complexes based on the H_2_L ligand have been prepared (Bai *et al.*, 2005; Wu *et al.*, 2004; Bai *et al.*, 2006; Milway *et al.*, 2003), but no complex containing rare earth elements has been reported to date. Herein, we report the structure of a new dinuclear Er^III^ complex (Scheme 1). The complex was synthesized by the 2:1:1 reaction of ErCl_3_.6H_2_O/α-pyridoin/salicylaldehyde thiosemicarbazone under solvothermal conditions. The X-ray analysis reveals that the centrosymmetric complex consists of two Er^III^ ions, two *L*^2-^ ligands, two Cl^-^ ions and two methanol molecules, Fig. 1 and Table 1. The intermolecular O—H···Cl hydrogen bonds, Table 2, lead to linear supramolecular chains along [010] (Fig. 2).

The remarkable structural feature of the complex is the presence of the *in situ* formed H_2_L ligand, which was proposed to be constructed by the reaction of picolinic acid, hydrazine and salicylaldehyde. The picolinic acid was assumed to be derived from the hydrolysis of α-pyridoin, and hydrazine and salicylaldehyde were believed to be originated from the hydrolysis of salicylaldehyde thiosemicarbazone (Narang & Aggarwal, 1974).

### Experimental

A mixture of ErCl_3_.6H_2_O (0.0762 g, 0.2 mmol), α-pyridoin (0.0214 g, 0.1 mmol), salicylaldehyde thiosemicarbazone (0.0390 g, 0.2 mmol) and CH_3_OH (2 ml) was sealed in a 6 ml Pyrex-tube. The tube was heated at 393 K for 3 days under autogenous pressure. Cooling of the resultant solution to room temperature gave yellow crystals. The crystals were collected by filtration, washed with CH_3_OH (2 ml) and dried in air.

### Refinement

The H atoms were placed in calculated positions with O—H = 0.95 Å and C—H = 0.95–0.98 Å, and with *U*_iso_(H) = 1.2–1.5*U*_eq_(C, O).

### Crystal data

**Table:**

[Er_2_(C_13_H_9_N_3_O_2_)_2_Cl_2_(CH_4_O)_2_]	*F*(000) = 908
*M**_r_* = 947.97	*D*_x_ = 2.089 Mg m^−^^3^
Monoclinic, *P*2_1_/*c*	Mo *K*α radiation, λ = 0.71073 Å
Hall symbol: -P 2ybc	Cell parameters from 4466 reflections
*a* = 9.5810 (4) Å	θ = 2.7–28.1°
*b* = 7.0906 (3) Å	µ = 5.76 mm^−^^1^
*c* = 22.3504 (8) Å	*T* = 128 K
β = 96.920 (3)°	Block, yellow
*V* = 1507.31 (10) Å^3^	0.15 × 0.13 × 0.12 mm
*Z* = 2	

### Data collection

**Table:**

Bruker SMART CCD diffractometer	3732 independent reflections
Radiation source: fine-focus sealed tube	2931 reflections with *I* > 2σ(*I*)
Graphite monochromator	*R*_int_ = 0.040
φ scans and ω scans	θ_max_ = 28.3°, θ_min_ = 2.1°
Absorption correction: multi-scan (*SADABS*; Bruker 2000)	*h* = −12→11
*T*_min_ = 0.479, *T*_max_ = 0.545	*k* = −9→9
14182 measured reflections	*l* = −29→29

### Refinement

**Table:**

Refinement on *F*^2^	Primary atom site location: structure-invariant direct methods
Least-squares matrix: full	Secondary atom site location: difference Fourier map
*R*[*F*^2^ > 2σ(*F*^2^)] = 0.027	Hydrogen site location: inferred from neighbouring sites
*wR*(*F*^2^) = 0.059	H-atom parameters constrained
*S* = 0.99	*w* = 1/[σ^2^(*F*_o_^2^) + (0.0299*P*)^2^] where *P* = (*F*_o_^2^ + 2*F*_c_^2^)/3
3732 reflections	(Δ/σ)_max_ = 0.004
200 parameters	Δρ_max_ = 1.25 e Å^−^^3^
0 restraints	Δρ_min_ = −0.71 e Å^−^^3^

### Special details

**Table:**

Geometry. All e.s.d.'s (except the e.s.d. in the dihedral angle between two l.s. planes) are estimated using the full covariance matrix. The cell e.s.d.'s are taken into account individually in the estimation of e.s.d.'s in distances, angles and torsion angles; correlations between e.s.d.'s in cell parameters are only used when they are defined by crystal symmetry. An approximate (isotropic) treatment of cell e.s.d.'s is used for estimating e.s.d.'s involving l.s. planes.
Refinement. Refinement of *F*^2^ against ALL reflections. The weighted *R*-factor *wR* and goodness of fit *S* are based on *F*^2^, conventional *R*-factors *R* are based on *F*, with *F* set to zero for negative *F*^2^. The threshold expression of *F*^2^ > σ(*F*^2^) is used only for calculating *R*-factors(gt) *etc*. and is not relevant to the choice of reflections for refinement. *R*-factors based on *F*^2^ are statistically about twice as large as those based on *F*, and *R*- factors based on ALL data will be even larger.

### Fractional atomic coordinates and isotropic or equivalent isotropic displacement parameters (Å^2^)

**Table:**

	*x*	*y*	*z*	*U*_iso_*/*U*_eq_	
Er1	0.372121 (17)	0.91591 (3)	0.432040 (7)	0.02740 (6)	
O2	0.5836 (3)	1.0615 (4)	0.46548 (11)	0.0323 (6)	
O3	0.2988 (3)	1.2290 (5)	0.42893 (15)	0.0526 (8)	
H7	0.3591	1.3150	0.4521	0.063*	
C5	0.6425 (4)	1.0757 (5)	0.36561 (16)	0.0289 (8)	
Cl1	0.48595 (13)	0.58790 (16)	0.42195 (5)	0.0476 (3)	
N1	0.5108 (3)	1.0104 (5)	0.34970 (14)	0.0282 (7)	
O1	0.1993 (3)	0.8698 (4)	0.36235 (12)	0.0386 (7)	
C14	0.0511 (4)	0.7742 (6)	0.46482 (18)	0.0366 (9)	
H14	−0.0122	0.7498	0.4935	0.044*	
C8	0.0727 (4)	0.7952 (5)	0.35398 (17)	0.0306 (9)	
C6	0.6814 (4)	1.1036 (5)	0.43082 (17)	0.0287 (8)	
N3	0.1753 (3)	0.8294 (5)	0.48577 (14)	0.0308 (7)	
N4	0.8066 (3)	1.1581 (5)	0.45048 (14)	0.0344 (8)	
C1	0.4695 (4)	0.9753 (6)	0.29156 (18)	0.0343 (9)	
H1	0.3765	0.9309	0.2802	0.041*	
C4	0.7340 (4)	1.1077 (6)	0.32293 (18)	0.0373 (10)	
H4	0.8257	1.1559	0.3347	0.045*	
C10	−0.1348 (5)	0.6619 (7)	0.3905 (2)	0.0435 (11)	
H10	−0.1858	0.6313	0.4230	0.052*	
C12	−0.1223 (5)	0.6715 (7)	0.2860 (2)	0.0440 (11)	
H12	−0.1625	0.6447	0.2459	0.053*	
C9	−0.0018 (4)	0.7457 (6)	0.40305 (17)	0.0332 (8)	
C3	0.6899 (5)	1.0688 (6)	0.26356 (19)	0.0413 (10)	
H3	0.7513	1.0886	0.2338	0.050*	
C13	0.0074 (4)	0.7590 (6)	0.29574 (18)	0.0366 (9)	
H13	0.0532	0.7954	0.2621	0.044*	
C2	0.5561 (5)	1.0008 (7)	0.24736 (18)	0.0393 (10)	
H2	0.5241	0.9721	0.2065	0.047*	
C11	−0.1934 (5)	0.6230 (7)	0.3332 (2)	0.0499 (12)	
H11	−0.2825	0.5629	0.3262	0.060*	
C7	0.1756 (6)	1.3080 (8)	0.3970 (3)	0.0722 (17)	
H7A	0.1078	1.2075	0.3850	0.108*	
H7B	0.2000	1.3728	0.3609	0.108*	
H7C	0.1341	1.3985	0.4229	0.108*	

### Atomic displacement parameters (Å^2^)

**Table:**

	*U*^11^	*U*^22^	*U*^33^	*U*^12^	*U*^13^	*U*^23^
Er1	0.02276 (9)	0.03156 (11)	0.02796 (10)	−0.00645 (8)	0.00334 (6)	−0.00213 (8)
O2	0.0269 (13)	0.0435 (18)	0.0273 (14)	−0.0087 (12)	0.0062 (10)	−0.0013 (12)
O3	0.0461 (19)	0.0366 (19)	0.073 (2)	0.0010 (15)	−0.0021 (15)	−0.0050 (17)
C5	0.0294 (18)	0.027 (2)	0.0304 (19)	−0.0045 (16)	0.0041 (15)	0.0008 (17)
Cl1	0.0544 (7)	0.0371 (6)	0.0535 (7)	0.0029 (5)	0.0145 (5)	0.0048 (5)
N1	0.0286 (16)	0.0276 (17)	0.0285 (17)	−0.0043 (13)	0.0038 (13)	−0.0008 (14)
O1	0.0266 (14)	0.054 (2)	0.0347 (16)	−0.0102 (13)	0.0012 (11)	−0.0015 (13)
C14	0.0283 (19)	0.042 (3)	0.040 (2)	−0.0070 (18)	0.0065 (16)	−0.003 (2)
C8	0.0251 (18)	0.032 (2)	0.034 (2)	0.0003 (15)	−0.0017 (15)	−0.0056 (17)
C6	0.0285 (19)	0.031 (2)	0.0273 (19)	−0.0042 (16)	0.0040 (14)	−0.0006 (16)
N3	0.0264 (16)	0.0361 (19)	0.0299 (17)	−0.0075 (14)	0.0033 (13)	−0.0026 (14)
N4	0.0288 (17)	0.046 (2)	0.0289 (18)	−0.0093 (15)	0.0066 (14)	−0.0029 (15)
C1	0.035 (2)	0.038 (3)	0.029 (2)	−0.0038 (17)	0.0004 (16)	0.0010 (18)
C4	0.032 (2)	0.045 (3)	0.035 (2)	−0.0083 (19)	0.0059 (16)	0.003 (2)
C10	0.035 (2)	0.048 (3)	0.046 (3)	−0.012 (2)	0.0013 (19)	−0.002 (2)
C12	0.036 (2)	0.047 (3)	0.046 (3)	−0.003 (2)	−0.0083 (19)	−0.011 (2)
C9	0.0263 (18)	0.036 (2)	0.036 (2)	−0.0036 (18)	0.0003 (15)	−0.0054 (19)
C3	0.041 (2)	0.049 (3)	0.036 (2)	0.002 (2)	0.0135 (18)	0.005 (2)
C13	0.033 (2)	0.040 (2)	0.036 (2)	0.0003 (19)	0.0012 (16)	−0.006 (2)
C2	0.044 (2)	0.047 (3)	0.026 (2)	0.000 (2)	−0.0002 (18)	0.0001 (19)
C11	0.037 (2)	0.050 (3)	0.060 (3)	−0.018 (2)	−0.007 (2)	−0.005 (2)
C7	0.069 (4)	0.058 (4)	0.088 (4)	0.019 (3)	0.003 (3)	0.013 (3)

### Geometric parameters (Å, º)

**Table:**

Er1—O1	2.157 (3)	C6—N4	1.286 (5)
Er1—O2^i^	2.284 (3)	N3—N4^i^	1.417 (4)
Er1—O2	2.316 (3)	N4—N3^i^	1.417 (4)
Er1—O3	2.327 (3)	C1—C2	1.376 (6)
Er1—N3	2.433 (3)	C1—H1	0.9500
Er1—N1	2.488 (3)	C4—C3	1.371 (6)
Er1—Cl1	2.5901 (12)	C4—H4	0.9500
O2—C6	1.320 (4)	C10—C11	1.362 (6)
O2—Er1^i^	2.284 (3)	C10—C9	1.403 (5)
O3—C7	1.419 (6)	C10—H10	0.9500
O3—H7	0.9500	C12—C11	1.369 (7)
C5—N1	1.351 (5)	C12—C13	1.382 (6)
C5—C4	1.390 (5)	C12—H12	0.9500
C5—C6	1.474 (5)	C3—C2	1.377 (6)
N1—C1	1.335 (5)	C3—H3	0.9500
O1—C8	1.316 (4)	C13—H13	0.9500
C14—N3	1.286 (5)	C2—H2	0.9500
C14—C9	1.427 (5)	C11—H11	0.9500
C14—H14	0.9500	C7—H7A	0.9800
C8—C13	1.398 (5)	C7—H7B	0.9800
C8—C9	1.423 (5)	C7—H7C	0.9800
			
O1—Er1—O2^i^	140.21 (10)	O1—C8—C13	120.5 (4)
O1—Er1—O2	149.99 (10)	O1—C8—C9	122.0 (3)
O2^i^—Er1—O2	66.16 (10)	C13—C8—C9	117.5 (3)
O1—Er1—O3	85.42 (12)	N4—C6—O2	124.5 (3)
O2^i^—Er1—O3	88.97 (11)	N4—C6—C5	119.5 (3)
O2—Er1—O3	80.42 (11)	O2—C6—C5	115.9 (3)
O1—Er1—N3	75.24 (10)	C14—N3—N4^i^	112.4 (3)
O2^i^—Er1—N3	65.42 (10)	C14—N3—Er1	129.4 (3)
O2—Er1—N3	130.77 (10)	N4^i^—N3—Er1	118.2 (2)
O3—Er1—N3	90.34 (11)	C6—N4—N3^i^	111.0 (3)
O1—Er1—N1	86.47 (10)	N1—C1—C2	122.7 (4)
O2^i^—Er1—N1	132.20 (10)	N1—C1—H1	118.6
O2—Er1—N1	66.06 (9)	C2—C1—H1	118.6
O3—Er1—N1	84.69 (11)	C3—C4—C5	119.0 (4)
N3—Er1—N1	161.38 (11)	C3—C4—H4	120.5
O1—Er1—Cl1	95.50 (9)	C5—C4—H4	120.5
O2^i^—Er1—Cl1	96.94 (7)	C11—C10—C9	122.4 (4)
O2—Er1—Cl1	93.86 (7)	C11—C10—H10	118.8
O3—Er1—Cl1	169.37 (9)	C9—C10—H10	118.8
N3—Er1—Cl1	100.15 (8)	C11—C12—C13	120.8 (4)
N1—Er1—Cl1	84.80 (8)	C11—C12—H12	119.6
O1—Er1—Er1^i^	167.32 (8)	C13—C12—H12	119.6
O2^i^—Er1—Er1^i^	33.34 (6)	C10—C9—C8	118.5 (4)
O2—Er1—Er1^i^	32.82 (6)	C10—C9—C14	117.5 (4)
O3—Er1—Er1^i^	83.65 (8)	C8—C9—C14	123.9 (3)
N3—Er1—Er1^i^	98.39 (7)	C4—C3—C2	119.6 (4)
N1—Er1—Er1^i^	98.87 (7)	C4—C3—H3	120.2
Cl1—Er1—Er1^i^	96.43 (3)	C2—C3—H3	120.2
C6—O2—Er1^i^	120.9 (2)	C12—C13—C8	121.5 (4)
C6—O2—Er1	124.5 (2)	C12—C13—H13	119.3
Er1^i^—O2—Er1	113.84 (10)	C8—C13—H13	119.3
C7—O3—Er1	128.4 (3)	C1—C2—C3	118.7 (4)
C7—O3—H7	115.8	C1—C2—H2	120.7
Er1—O3—H7	115.8	C3—C2—H2	120.7
N1—C5—C4	121.5 (4)	C10—C11—C12	119.2 (4)
N1—C5—C6	115.0 (3)	C10—C11—H11	120.4
C4—C5—C6	123.5 (3)	C12—C11—H11	120.4
C1—N1—C5	118.5 (3)	O3—C7—H7A	109.5
C1—N1—Er1	123.2 (3)	O3—C7—H7B	109.5
C5—N1—Er1	117.6 (2)	H7A—C7—H7B	109.5
C8—O1—Er1	141.0 (2)	O3—C7—H7C	109.5
N3—C14—C9	126.9 (4)	H7A—C7—H7C	109.5
N3—C14—H14	116.5	H7B—C7—H7C	109.5
C9—C14—H14	116.5		

Symmetry code: (i) −*x*+1, −*y*+2, −*z*+1.

### Hydrogen-bond geometry (Å, º)

**Table:**

*D*—H···*A*	*D*—H	H···*A*	*D*···*A*	*D*—H···*A*
O3—H7···Cl1^ii^	0.95	2.42	3.128 (4)	131

Symmetry code: (ii) *x*, *y*+1, *z*.

## References

[bb1] Bai, Y., Dang, D. B., Cao, X., Duan, C. Y. & Meng, Q. J. (2006). *Inorg. Chem. Commun.* **9**, 86–89.

[bb2] Bai, Y., Dang, D. B., Duan, C. Y., Song, Y. & Meng, Q. J. (2005). *Inorg. Chem.* **44**, 5972–5974. 10.1021/ic0506254 16097812

[bb3] Bruker (2000). *SADABS*, *SAINT* and *SMART* Bruker AXS Inc., Madison, Wisconsin, USA.

[bb4] Guo, Y. N., Chen, X. H., Xue, S. F. & Tang, J. K. (2011*a*). *Inorg. Chem.* **50**, 9705–9713. 10.1021/ic2014978 21902183

[bb5] Guo, Y. N., Xu, G. F., Wernsdorfer, W., Ungur, L., Guo, Y., Tang, J. K., Zhang, H. J., Chibotaru, L. F. & Powell, A. K. (2011*b*). *J. Am. Chem. Soc.* **133**, 11948–11951. 10.1021/ja205035g 21744848

[bb6] Milway, V. A., Zhao, L., Abedin, T. S. M., Thompson, L. K. & Xu, Z. Q. (2003). *Polyhedron*, **22**, 1271–1279.

[bb7] Narang, K. K. & Aggarwal, A. (1974). *Inorg. Chim. Acta*, **9**, 137–142.

[bb8] Sheldrick, G. M. (2008). *Acta Cryst.* A**64**, 112–122. 10.1107/S0108767307043930 18156677

[bb9] Wu, W. S., Feng, Y. L., Lan, X. R. & Huang, T. T. (2004). *Chin. J. Appl. Chem.* **21**, 135–139.

